# Could early infusion of fish-oil-based lipid emulsion affect the need for intensive care in moderately diseased COVID-19 patients? A randomized clinical trial

**DOI:** 10.1186/s42077-022-00251-0

**Published:** 2022-07-16

**Authors:** Omar M. Soliman, Yara H. Abbas, Arafa Mohamed Ahmed Aboelhassan, Eman Ahmed Ismail

**Affiliations:** 1 Department of Anesthesia and Intensive Care, Faculty of Medicine, Assiut University, Assiut, Egypt; 2 Department of Pulmonology, Faculty of Medicine, Assiut University, Assiut, Egypt

**Keywords:** COVID-19, Enteral nutrition, Fish-oil-based lipid emulsion, Severe acute respiratory syndrome coronavirus 2 (SARS-COV-2), Shift to ICU, SMOF lipid 20%

## Abstract

**Background:**

Sixty moderate diseased COVID-19 patients were divided into two equal groups and were enrolled in a randomized double**-**blind clinical trial. Group C was delivered standard enteral nutrition plus 100 ml/day of 0.9% normal saline. Group L was delivered fish-oil-based lipid emulsion (FOBLE) supplementation to standard enteral nutrition at a dose of 100 ml/day. Both groups infused at a rate of 12.5 ml/h over 8 h for 5 days. We aimed to compare the effect of FOBLE versus placebo in COVID-19 disease to clarify the impact on the number of patients shifted to the ICU, oxygenation, inflammatory markers, and short-term outcomes (7 days).

**Results:**

The failed conventional care and shift to ICU was significantly lower in group L in comparison to group C (six patients (20.0%) versus 14 patients (46.7%) shifted to ICU, *P*-value = 0.028). The inflammatory markers were determined and evaluated. Throughout the trial, there were no significant changes with the exception of the 7th day neutrophil/lymphocyte ratio (NLR), when the ratio was lower in group L than in group C (6.10 (3.90–7.20) versus 9.65 (8.30–10.90), respectively, *P*-value 0.001).

**Conclusions:**

In moderate diseased COVID-19 patients, early administration of parenteral FOBLE as an adjuvant to enteral feeding reduces shifts and so minimizes the burden on the ICU.

**Trial registration:**

ClinicalTrials.gov PRS (NCT04957940). Registered on 5 July 2021.

**Supplementary Information:**

The online version contains supplementary material available at 10.1186/s42077-022-00251-0.

## Background

Severe acute respiratory syndrome coronavirus 2 (SARS-CoV-2) is a novel coronavirus discovered in Wuhan, China, in December 2019 (Gorbalenya et al. 2020). COVID-19 was declared a pandemic by the World Health Organization on March 11, 2020 (WHO 2020).

Antiviral medications like lopinavir, ritonavir, and remdesivir; antibacterial treatments like macrolides; and antimalarial drugs like hydroxychloroquine are used to treat COVID-19. The major approach is to optimize respiratory functions, especially in those with lower respiratory tract involvement. Supportive therapy appears to be the most successful treatment method during the course of the disease. The condition has a greater morbidity and mortality rate, particularly in elderly patients with weakened immune systems, those with dietary deficits, and those with chronic illnesses (Hu et al. 2017; Mehta, 2020).

A fish-oil-based lipid emulsion (FOBLE) is a product that is high in omega-3 fatty acids, such as eicosapentaenoic acid (EPA) and docosahexaenoic acid (DHA), which are long-chain polyunsaturated fatty acids. Heller et al. (2006) have shown that supplementation with FOBLE or Ꞷ-3 fatty acids improves outcomes in various patient subgroups.

We believe that administering FOBLE early in the moderate cases of COVID-19 alters the inflammatory response and decreases the number of patients shifted to the ICU.

## Methods

This was a randomized, double-blind, controlled clinical trial, approved by the University’s Institutional Review Board (IRB17300631 on July 2021). The methodology of this study followed the Declaration of Helsinki (revised DOH 2013). Written informed consent was taken from the patients or their 1st-degree relatives. The trial was registered before patient enrollment in the ClinicalTrials.gov (NCT04957940) in July 2021.

Patients who were ≥ 18 years with the diagnosis of moderate cases of COVID-19 and a person who had a lower respiratory illness, such as pneumonia, were enrolled. However, their oxygen saturation levels remained ≥ 94% (National Institute of Health 2021) requiring only conventional therapy (supplemental oxygen in the form of a simple nasal cannula or venturi mask oxygen, anti-viral, antipyretic medications) with good enteral nutrition. Diagnosis of the cases was confirmed using RT-PCR for detection of the viral RNA by TaqMan™ 2019-nCoV Control Kit v1 (Cat. No. A47532) supplied by QIAGEN, Germany on the Applied Biosystem 7500 Fast RT PCR System, USA. Patients were excluded with severe criteria of COVID-19 before the five doses of SMOF lipid 20% were completed, hypersensitivity, uncontrolled hyperlipidemia, severe primary blood coagulation diseases, acute pancreatitis, acute thromboembolic diseases, severe liver failure, RIFLE stage III and IV renal failure, pregnancy or lactation, and severe neutropenia (< 500 cells/mm3).

Patients who met the enrollment requirements were assigned an ascending serial number in the sequence in which they were enrolled. The patients were assigned to one of the trial groups based on this number. The patient’s prescription included a patient number, which was utilized to prepare the all-in-one bag for the patient. The clinical pharmacist assisted in the creation of the double-blind solutions (by covering the solution bottle and connecting lines by the opaque shield). Study masking: outcome assessors and patients were unaware of the randomization until all subjects’ 7-day (within the study period) follow-ups were completed.

At the time of admission, patients had a peripheral venous line placed. Patients were randomly assigned to receive standard enteral nutrition plus 100 ml/day of 0.9% normal saline at a rate of 12.5 ml/h over 8 h for 5 days as the control group (Group C) or intravenous fish-oil-based lipid (SMOF lipid 20%) emulsion supplementation to standard enteral nutrition (group L) in a dose of 100 ml/day at a rate of 12.5 ml/h over 8 h for 5 days. To flush the system, 20 mL of 0.9% normal saline was injected into the burette. When each infusion was finished, the entire equipment was discarded. For each infusion, the entire procedure was repeated.

The neutrophil/lymphocyte ratio (NLR), serum C-reactive protein (CRP) levels, lactate, D-dimer, serum ferritin (recorded at 0, 2, 5, and 7 days), and interleukin-6 (IL-6) levels (recorded at 0 and day 7) were measured. Sodium, potassium, chloride, AST, ALT, total triglycerides, random blood sugar (RBS), urea, creatinine, albumin, and total bilirubin were all measured (recorded at 0 and day 7). Partial arterial oxygen tension/ fraction of inspired oxygen PaO2/ FiO2 (P/F) ratio and respiratory rate (RR) were assessed and recorded at 0, 2, 3, 5, and 7 days.

Comorbidities, e.g., DM, hypertension, and obstructive sleep apnea, were recorded and patients continued with their regular treatment. Failed conventional care treatment, converted patients to severe cases, and shift to the ICU for more advanced treatment (more oxygenation and/or ventilation) were recorded. Severe illness was defined for individuals who had SpO2 < 94% on room air at sea level, a ratio of arterial partial pressure of oxygen to fraction of inspired oxygen (PaO2/FiO2) < 300, respiratory frequency > 30 breaths per minute, or lung infiltrates > 50% (National Institute of Health 2021), taking consideration that P/F ratio for severe acute respiratory distress syndrome (ARDS) is < 100.

The primary outcome was the number of patients shifted to the ICU for upgrading oxygenation and/or ventilation. Secondary outcomes were short-term outcome (7 days), P/F ratio, RR, NLR, serum CRP, lactate, D-dimer, ferritin, and interleukin-6 levels. Adverse events or complications related to SMOF lipid 20% were treated and recorded.

### Sample size

This study was a randomized trial to evaluate the impact of FOBLE on the number of patients shifted to the ICU for upgrading oxygenation and/or ventilation. Sample size calculation was carried out using G*Power 3 software. A calculated minimum sample of 52 participants was needed to be divided into two equal groups in order to detect an effect size of 0.8 with an error probability of 0.05 and 80% power on a two-tailed test. We added 15% to the calculated sample to compensate for dropouts.

### Statistical analysis

**Fig. 1:**
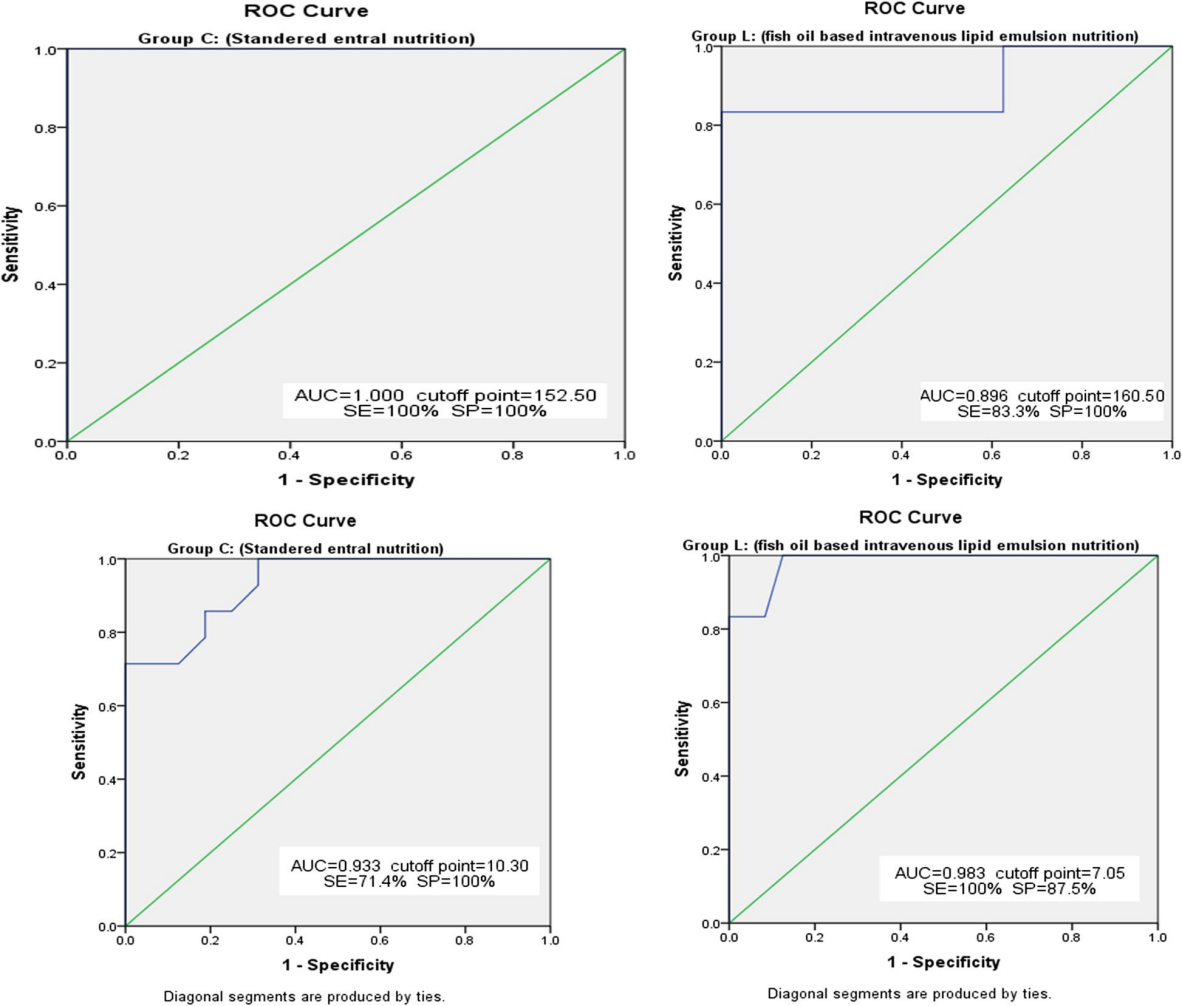
The 7th-day P/F & NLR ROC curves and their correlation in predicting failed conventional care and shift to ICU between the studied groups

SPSS version 22.0 was used for data management and data analysis. Numbers with percentages described qualitative data. The chi-square test and Fisher’s exact test were used for comparing independent categorical variables. Statistical analysis of the data was conducted for baseline results. We presented descriptive statistics (e.g., mean and standard deviation for normally distributed variables, or median and interquartile range for skewed variables). Differences between groups were analyzed using the Student *t*-test or Mann–Whitney test for normally distributed or skewed variables respectively. ROC curve analysis of 7th day P/F and NLR was used in predicting failed conventional care and shift to ICU (Fig. 1). The *P*-value was two-tailed and considered significant at 0.05 levels.

## Results

**Fig. 2:**
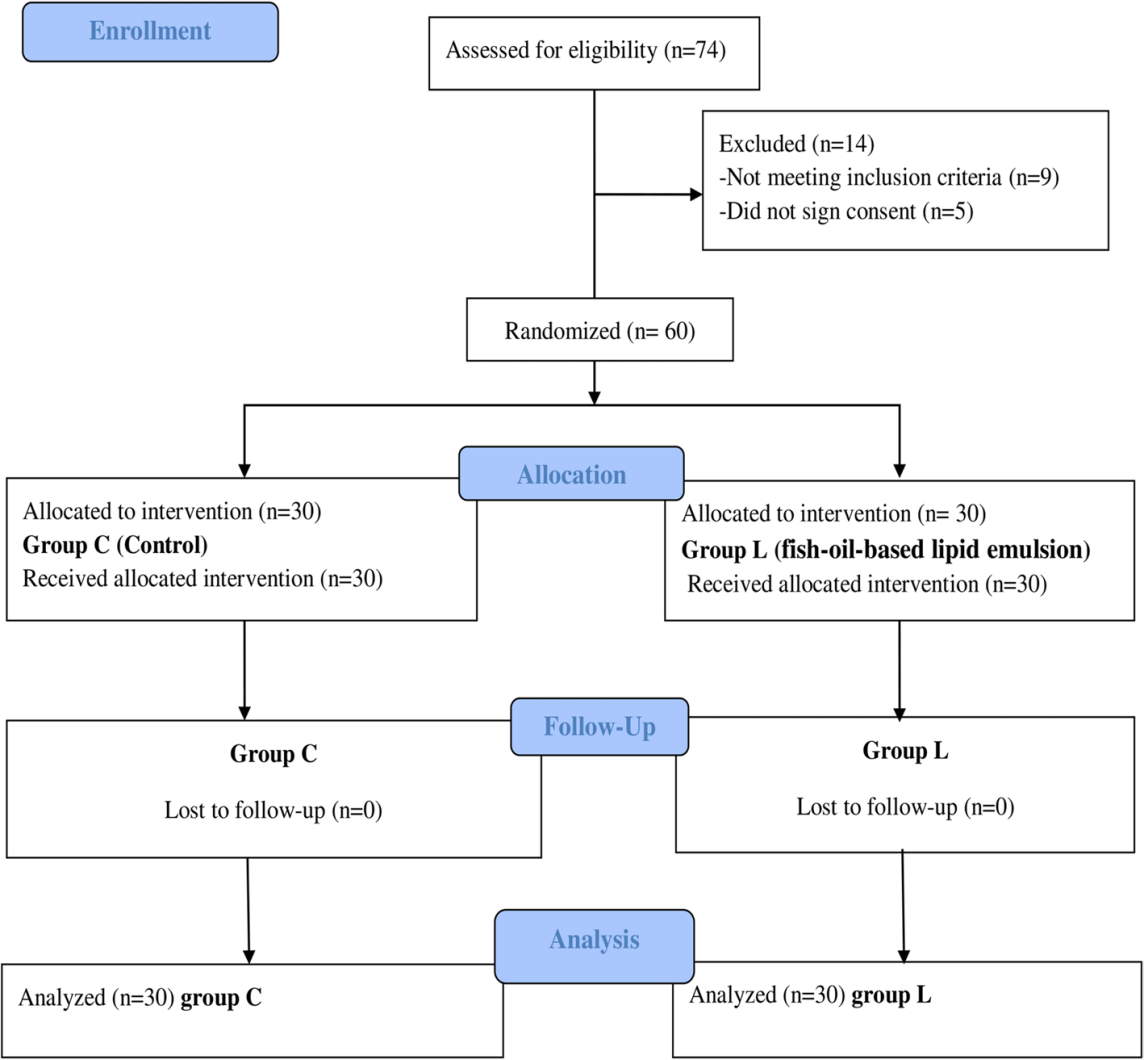
CONSORT Flow Chart between the study group

Among the 74 patients who were screened for eligibility, 14 patients were excluded (9 did not meet inclusion criteria, 5 patients did not sign for consent). Sixty patients were finally analyzed between the two study groups, as shown in the flow chart of the studied groups (Fig. 2). The demographic data of the enrolled patients (age, gender, weight, height, body mass index (BMI), and the clinical data (smoking, and co-existing diseases) showed no significant differences between the two study groups (Table 1). Also, there were no significant differences between both groups regarding laboratory data (HB, WBCs, platelets, random blood sugar, total triglycerides) as in Supplementary table 1 and (Na, K, Cl, AST, ALT, albumin, total proteins, urea, creatinine) as shown in Supplementary table 2.

**Table 1:** Demographic and clinical data in the two study groups

Variables	Group C (*n* = 30)	Group L (*n* = 30)	*P*-value
**Age (years)**	61.50 ± 6.79	61.10 ± 6.56	0.817
**Gender (m/f)**	19/11 (63.3/36.7%)	21/9 (70.0/30.0%)	0.584
**Weight (kg)**	91.30 ± 7.01	91.47 ± 8.45	0.934
**Height (cm)**	168.03 ± 4.90	168.57 ± 4.94	0.676
**Body mass index (kg/m**^**2**^**)**	32.37 ± 2.48	32.22 ± 2.96	0.832
**Smoking (No/Yes)**	13/17 (43.3/56.7%)	14/16 (46.7/53.3%)	0.795
**Co-existing diseases**			
**NO**	9 (30.0%)	8 (26.7%)	0.975
**HTN**	4 (13.3%)	3 (10.0%)	
**DM**	2 (6.7%)	2 (6.7%)	
**DM and HTN**	4 (13.3%)	3 (10.0%)	
**Other combined diseases**	11 (36.7%)	13 (43.3%)	
**Oncology**	0 (0.0%)	1 (3.3)	

Data are presented as mean ± standard deviation, number (percentage)

*HTN* Hypertension, *DM* Diabetes mellitus

Group C (standard enteral nutrition) and Group L (fish-oil-based intravenous lipid emulsion)

*P* < 0.05 is considered statistically significant

**Fig. 3:**
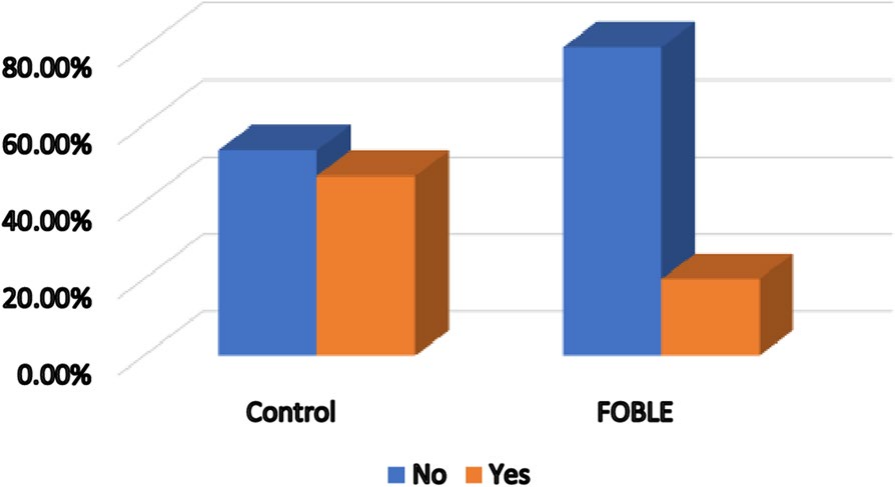
Failed conventional care and shift to ICU between the two groups

Regarding the failed conventional care and shift to ICU, it was significantly lower in group L in comparison to group C (six patients (20.0%) versus 14 patients (46.7%) shifted to ICU, *P*-value = 0.028) as shown in Fig. 3.

The inflammatory markers were measured and assessed. There were no significant differences between the studied groups for serum ferritin, D-dimer, serum lactate, CRP, IL-6 levels, and NLR throughout the study time, except for the 7th day NLR, where the ratio was decreased in group L than in group C (6.10 (3.90–7.20) versus 9.65 (8.30–10.90) respectively, *P*-value < 0.001) as shown in Table 2.

**Table 2:** Inflammatory markers in the two study groups

Variables	Group C (*n* = 30)	Group L (*n* = 30)	*P*-value
NLR			
Admission	5.25 (3.30–8.40)	5.60 (3.60–9.00)	0.564
2nd day	6.35 (3.70–8.30)	6.05 (4.40–8.70)	0.813
5th day	6.75 (5.20–8.40)	5.85 (8.40–6.80)	0.130
7th day	9.65 (8.30–10.90)	6.10 (3.90–7.20)	< 0.001*
Serum ferritin ng/ml			
Admission	200.50 (130.00–320.00)	264.50 (128.00–322.00)	0.712
2^nd^ day	231.00 (180.00–322.00)	292.50 (190.00–331.00)	0.679
5th day	289.00 (193.00–357.00)	276.50 (220.00–329.00)	0.767
7th day	335.50 (202.00–421.00)	285.50 (221.00–352.00)	0.344
D-dimer mcg/ml			
Admission	1.35 (0.80–2.20)	1.15 (0.80–2.00)	0.784
2nd day	1.50 (1.00–2.40)	1.35 (0.90–1.90)	0.424
5th day	1.90 (1.20–2.50)	1.30 (1.10–2.00)	0.058
7th day	2.05 (1.10–3.50)	1.20 (0.90–2.10)	0.087
Serum lactate mmol/L			
Admission	1.30 (0.90–2.00)	1.20 (0.80–2.00)	0.568
2nd day	1.20 (1.00–1.60)	1.10 (1.00–1.70)	0.242
5th day	1.35 (1.10–1.90)	1.20 (1.00–2.00)	0.509
7th day	1.50 (1.10–2.40)	1.10 (0.90–1.80)	0.073
CRP mg/dl			
Admission	15.50 (13.00–24.00)	17.50 (12.00–25.00)	0.871
2nd day	20.50 (15.00–26.00)	18.00 (14.00–24.00)	0.549
5th day	22.00 (13.00–32.00)	18.00 (13.00–23.00)	0.131
7th day	21.50 (15.00–44.00)	17.50 (13.00–22.00)	0.110
IL-6 level pg/ml			
Admission	7.13 ± 2.75	6.91 ± 2.99	0.764
7th day	7.80 (6.30–10.70)	7.40 (6.00–8.60)	0.297

Data are presented as median and IQ (interquartile range) and mean ± SD

*NLR* Neutrophil/lymphocyte ratio, *CRP* C-reactive protein

Group C (standard enteral nutrition) and Group L (fish-oil-based intravenous lipid emulsion)

^*^P < 0.05 is considered statistically significant

Regarding RR, there was less increase in group L in comparison to group C on the 5th and 7th day (5th day RR of 15.00 versus 18.00 respectively, *P*-value = 0.005, 7th day RR of 18.00 versus 20.00 respectively, *P*-value = 0.017). For P/F ratio, there was less decrease in group L than in group C (5th day P/F ratio 198.50 (188.00–216.00) versus 182.50 (123.00–215.00) respectively, *P*-value = 0.044, 7th day P/F ratio 197.50 (178.00–215.00) versus 169.50 (100.00–203.00) respectively, *P*-value = 0.019) as shown in Table 3.

**Table 3:** Oxygenation and ventilation variables, respiratory rate (RR), and outcome in the two study groups

Variables	Group C (*n* = 30)	Group L (*n* = 30)	*P*-value
**Need for ventilation and/or oxygenation**			
**No need**	16 (53.3%)	24 (80.0%)	0.028*
**In need**	14 (46.7%)	6 (20.0%)	
**Respiratory rate (RR)**			
**Admission**	14.00	14.00	0.705
**2nd day**	15.00	14.00	0.087
**3rd day**	16.00	14.00	0.224
**5th day**	18.00	15.00	0.005*
**7th day**	20.00	18.00	0.017*
**P/F ratio**			
**Admission**	211.97 ± 26.78	206.70 ± 29.52	0.472
**2nd day**	203.70 ± 30.14	204.07 ± 26.67	0.960
**3rd day**	190.27 ± 35.40	202.17 ± 27.71	0.152
**5th day**	182.50 (123.00–215.00)	198.50 (188.00–216.00)	0.044*
**7th day**	169.50 (100.00–203.00)	197.50 (178.00–215.00)	0.019*
**Outcome**			
**Home discharge**	3	8	0.055
**ICU shift**	14	6	
**Continued treatment inward after a study time frame**	13	16	
**Died**	0	0	

Data are presented as numbers (percentages)

*P/F ratio* Partial arterial oxygen tension/faction of inspired oxygen

Group C (standard enteral nutrition) and Group L (fish-oil-based intravenous lipid emulsion)

^*^P < 0.05 is considered statistically significant

The ROC curves for the 7th day P/F and NLR and their correlation in predicting failed conventional care and shift to ICU between the studied groups showed good to excellent diagnostic tests. ROC curves for 7th day P/F showed a higher area under the ROC curve in group C in correlation to group L (1 versus 0.896). The optimal cut-off points were 152.50 in group C at sensitivity (SE) = 100% and specificity (SP) = 100% versus 160.50 in group L at SE = 83.3% and SP = 100%. ROC curves for 7th day NLR showed a higher area under the ROC curve in group L than in group C (0.983 versus 0.933). The optimal cut-off points were 7.05 in group L at SE = 100% and SP = 87.5% versus 10.30 in group C at SE = 71.4% and SP = 100% as shown in Fig. 1.

The outcome was compared between both groups; home discharge (eight patients in group L versus three in group C), ICU shift (six patients in group L versus 14 in group C), and continued conventional treatment after the study time frame (16 patients in group L versus 13 in group C), and no patients died throughout the study time (7 days), *P*-value = 0.055 as shown in Table 3.

## Discussion

Modulating the inflammatory response in SARS-CoV-2 is one of the target therapies. This study investigated the early effect of intravenous FOLE in moderate diseased COVID-19 patients to reduce the burden of inflammatory mediators and reduce the need for ICU. All COVID-19 patients were treated according to management protocol for COVID-19 patients Ministry of Health (Masoud et al. 2020).

In our trial, we found that the number of patients shifted to ICU was lower in the group that received FOBLE in comparison to the group that received standard enteral nutrition. Also, by the 5th and 7th days, the inflammatory response was reduced, and oxygenation was less affected in patients who received FOBLE supplemented enteral nutrition.

When SARS-CoV-2 infects the upper and lower respiratory tracts, it appears to activate a cascade of inflammation in the lower respiratory tract, resulting in ARDS (Rabi et al 2020). COVID-19 severity is linked to a cytokine profile comparable to secondary hemophagocytic lympho-histiocytosis, as well as high levels of ferritin and IL-6, both of which are considered mortality predictors (Mehta et al. 2020). This could be a sign of viremia-induced hyperinflammation.

ω-3 oral, enteral, or parenteral administration of polyunsaturated fatty acids (PUFAs) is possible. When administered intravenously (1–3 days), they are absorbed into plasma phospholipids and blood cells faster than when given orally or enterally (4–7 days) (van der Meij et al. 2011). PUFAs help membrane phospholipids maintain their structural integrity and fluidity. Furthermore, PUFAs impact gene expression and serve as a source of lipid mediators such as eicosanoids (Calder and Grimble 2002). The omega-6 fatty acid arachidonic acid (ARA), as well as the omega-3 fatty acids eicosapentaenoic acid (EPA) and docosahexaenoic acid (DHA), influences inflammatory and immunological responses. Eicosanoids generated from EPA and DHA are often less inflammatory than ARA-derived eicosanoids. EPA-derived mediators, for example, have a lower affinity for eicosanoid receptors than ARA-derived mediators (Calder and Yaqoob 2012).

The anti-inflammatory impact of EPA and DHA has been linked to a decrease in ARA in membrane phospholipids, which results in less inflammatory ARA-derived lipid mediators being produced and more inflammatory EPA-derived lipid mediators being produced (Calder 2020). Supplementing with EPA and DHA can boost the proportion of both fatty acids in blood lipids, blood cells, and a variety of organ compartments. EPA and DHA are incorporated into phospholipids in a dose and time-dependent manner, with EPA being incorporated faster than DHA (Calder 2012).

SMOF lipid is a mix of coconut oil, soybean oil, olive oil, and fish oil. Fish oil is high in bioactive ω-3 polyunsaturated fatty acids (EPA and DHA), which suppress the synthesis of pro-inflammatory eicosanoids and cytokines while promoting the creation of anti-inflammatory cytokines and inflammation-resolving lipid mediators (Calder 2009).

In line with our study, Weill et al. (2020) examined the features of ω-3 PUFAs, which include interference with viral entry and replication as well as inflammatory inhibition, resulting in improved outcomes in critically sick patients with ARDS. When bronchoalveolar lavage fluid was added to A549 cells, it was discovered that increasing the ratio of ω-3:ω-6 PUFAs resulted in lower levels of nuclear factor kappa B (NF-kB), cyclooxygenase-2 (COX-2), and prostaglandin E2 (PGE2), as well as higher levels of IL-10 and peroxisome proliferator-activated receptor gamma (PPAR) (Cotogni et al. 2015).

Inflammatory mediators are reduced in the bronchoalveolar lavage fluid of patients with acute respiratory distress syndrome after administration of ω-3 fatty acids, according to Pacht et al. (2003). In a dose-dependent way, Heller et al. (2006) found that parenteral fish oil emulsion boosted survival while decreasing infection rates, antimicrobial needs, and duration of hospital stay.

Several experimental results have been made public. Hecker et al. (2014) looked at how three parenteral lipid emulsions affected morphology, leukocyte infiltration, protein leakage, and cytokine production in a mouse model of ARDS. Fish oil was found to have anti-inflammatory and pro-resolving effects on lung injury when compared to long-chain triglycerides (LCT) or medium-chain triglycerides (MCT)/LCT emulsions, indicating that partial replacement of n-6 fatty acids with marine n-3 fatty acids during ARDS may be beneficial. In a rat model of acute lung injury, Kohama et al. (2014) found that, when compared to soybean oil, parenteral nutrition supplemented with fish oil improved gas exchange and inhibited both neutrophil recruitment and upregulation of inflammatory mediator mRNAs, as well as increased anti-inflammatory eicosanoid 4-series leukotrienes (LTB5) production.

In addition, Hosny et al. (2013) studied the efficacy and safety of high-dose EPA and DHA supplementation (9 g/d with 1 g/day ascorbic acid + 400UI/12 h alpha-tocopherol and 100 g/day selenium) in patients with early-stage sepsis for 7 days. CRP, IL-6, and procalcitonin levels were found to be lower in the supplemented group than in the control group. In addition to these findings, the supplemented group had a lower requirement for mechanical breathing, a shorter period of mechanical ventilation, and a lower risk of developing severe sepsis, according to the authors.

In contrast, a randomized multicenter prospective study in patients with acute lung injury comparing intermittent enteral fish oil (EPA and DHA) to a normal saline bolus over 14 days found no benefit in inflammatory biomarkers in bronchoalveolar lavage and plasma, nor improvement in organ failure score, ventilator-free days, intensive care unit-free days, or 60-day mortalities (Stapleton et al. 2011).

In a prospective cross-over investigation of 19 patients with early ARDS (3 ± 2 days after onset of ARDS, after at least 12 h of stability of PaO2/FiO2 without any change in ventilator settings), mixed emulsions including MCTs and LCTs were found to have no effect on gas exchange (Smyrniotis et al. 2001).

### Points of strengths

The study’s strengths were the fact that the two groups were assigned at random and that nearly all of the patients in both groups had co-existing illnesses. We could also add SMOF lipid 20% as a supplement because we provided FOBLE early after admission and our trial comprised of patients who had good enteral nutrition.

### Limitations

Our findings cannot be generalized to all COVID-19 patients, particularly those who are severely ill. The study was limited in that it only looked at moderate cases of COVID-19 patients, with severe cases being eliminated. Second, we did not follow up with patients after the study ended, thus we were unable to determine their final outcomes. Finally, large size multicenter studies are required to reach this conclusion.

### Recommendations

Our findings suggest that early use of a fish-oil-based lipid emulsion could minimize the inflammatory response and enhance the outcome in moderate cases of COVID-19. To better understand the impact of this promising diet, we urge that researchers do trials on varied concentrations and timings of FOBLE in different stages of COVID-19.

## Conclusions

This trial shows that early administration of parenteral FOBLE (SMOF lipid 20%) as an adjuvant treatment to enteral nutrition reduces transfers to the ICU by lowering inflammation, preventing oxygenation deterioration, and improving prognosis in COVID-19 patients with moderate disease.

## Supplementary Information

**Additional file 1:** **Supplemental Table 1.** First laboratory markers in the two study groups.

**Additional file 2:** **Supplemental Table 2.** Second laboratory markers in the two study groups.

## Data Availability

The datasets used and/or analyzed during the current study are available from the corresponding author on reasonable request.

## Abbreviations

COVID-19: Coronavirus disease of 2019
FOBLE: Fish-oil-based lipid emulsion
SARS-COV-2: Severe acute respiratory syndrome coronavirus 2
SE: Sensitivity
SP: Specificity
EPA: Eicosapentaenoic acid
DHA: Docosahexaenoic acid
NLR: Neutrophil/lymphocyte ratio
CRP: C-reactive protein
IL-6: Interleukin-6
RBS: Random blood sugar
P/F: PaO2/ FiO2 ratio
RR: Respiratory rate
ARDS: Severe acute respiratory distress syndrome
BMI: Body mass index
PUFAs: Polyunsaturated fatty acids
ARA: Acid arachidonic acid
NF-kB: Nuclear factor kappa B
COX-2: Cyclooxygenase-2
PGE2: Prostaglandin E2
PPAR: Peroxisome proliferator-activated receptor gamma
LCT: Long-chain triglycerides
MCT: Medium-chain triglycerides
