# Supplementary material for: Could early infusion of fish-oil-based lipid emulsion affect the need for intensive care in moderately diseased COVID-19 patients? A randomized clinical trial
Source: Ain-Shams J Anesthesiol. 2022 Jul 16;14(1):54. doi: 10.1186/s42077-022-00251-0 (PMC9287710; doi:10.1186/s42077-022-00251-0)
Supplement: Supplementary file 1 — Additional file 1: Supplemental Table 1. First laboratory markers in the two study groups. [file 42077_2022_251_MOESM1_ESM.docx]

| **Variables** | **Group C**  **(n=30)** | **Group L**  **(n=30)** | ***P*-value** |
| --- | --- | --- | --- |
| **HB (g/dL)**  **Admission**    **7th day** | 10.95 (10.00-12.20) | 11.25 (10.20-12.30) | 0.451 |
|  | 10.00 (9.60-11.40) | 10.45 (10.00-11.20) | 0.178 |
| **WBCs**  **Admission**    **7th day** | 9.60 (7.60-10.50) | 9.20 (7.70-10.50) | 0.853 |
|  | 9.95 (8.90-11.20) | 9.60 (7.00-11.10) | 0.267 |
| **Platelets**  **Admission**    **7th day** | 315.50 (234.00-391.00) | 300.50 (274.00-370.00) | 0.712 |
|  | 296.50 (249.00-356.00) | 309.00 (264.00-355.00) | 0.652 |
| **RBS (mg/dL)**  **Admission**    **7th day** | 130.00 (109.00-175.00) | 128.50 (102.00-174.00) | 0.723 |
|  | 135.50 (119.00-171.00) | 126.00 (118.00-164.00) | 0.412 |
| **Total Triglycerides**  **Admission**    **7th day** | 113.57**±**18.50 | 112.27**±**18.03 | 0.784 |
|  | 124.07**±**21.26 | 122.47**±**21.32 | 0.772 |

**Supplemental Table (1): First Laboratory Markers in the two study groups**

Data are presented as median & IQ=interquartile range and mean ±SD. HB (hemoglobin), WBCs (white blood cells), RBS (random blood sugar), (*) *P*<0.05 is considered statistically significant. Group C (standard enteral nutrition) and Group L (fish-oil-based intravenous lipid emulsion).
