# Supplementary material for: Could early infusion of fish-oil-based lipid emulsion affect the need for intensive care in moderately diseased COVID-19 patients? A randomized clinical trial
Source: Ain-Shams J Anesthesiol. 2022 Jul 16;14(1):54. doi: 10.1186/s42077-022-00251-0 (PMC9287710; doi:10.1186/s42077-022-00251-0)
Supplement: Supplementary file 2 — Additional file 2: Supplemental Table 2. Second laboratory markers in the two study groups. [file 42077_2022_251_MOESM2_ESM.docx]

**Supplemental Table (2): Second Laboratory Markers in the two study groups**

| **Variables** | **Group C (n=30)** | **Group L (n=30)** | ***P*-value** |
| --- | --- | --- | --- |
| **Na (mEq/L)** | | | |
| **Admission** | 131.67±5.63 | 131.73±5.67 | 0.964 |
| **7^th^ day** | 136.17±6.08 | 136.00±5.81 | 0.914 |
| **K (mEq/L)** | | | |
| **Admission** | 4.14±0.57 | 4.08±0.62 | 0.667 |
| **7^th^ day** | 4.33±0.48 | 4.17±0.40 | 0.186 |
| **CL (mEq/L)** | | | |
| **Admission** | 94.27**±**5.49 | 95.50**±**5.08 | 0.370 |
| **7^th^ day** | 96.07**±**3.81 | 94.37**±**3.48 | 0.076 |
| **Albumin (g/L)** | | | |
| **Admission** | 39.23±5.69 | 38.47±6.00 | 0.614 |
| **2^nd^ day** | 35.97±5.10 | 37.97±4.68 | 0.119 |
| **Total proteins (g/L)** | | | |
| **Admission** | 73.81±5.56 | 72.84±6.52 | 0.538 |
| **7^th^ day** | 69.49±6.98 | 72.67±6.75 | 0.078 |
| **AST (U/L)** | | | |
| **Admission** | 27.50 (20.00-31.00) | 27.50 (19.00-33.00) | 0.722 |
| **7^th^ day** | 30.00 (18.00-37.00) | 23.00 (18.00-35.00) | 0.549 |
| **ALT (U/L)** | | | |
| **Admission** | 25.00 (16.00-29.00) | 24.00 (18.00-32.00) | 0.778 |
| **7^th^ day** | 29.00 (15.00-37.00) | 21.00 (15.00-31.00) | 0.321 |
| **Urea (mg/dL)** | | | |
| **Admission** | 16.50 (12.00-22.00) | 18.00 (13.00-23.00) | 0.569 |
| **7th day** | 20.00 (11.00-29.00) | 17.00 (11.00-22.00) | 0.366 |
| **Creatinine (umol/L)** | | | |
| **Admission** | 77.00 (63.00-93.00) | 80.00 (68.00-98.00) | 0.246 |
| **7^th^ day** | 74.00 (58.00-91.00) | 79.00 (64.00-89.00) | 0.492 |

Data are presented as median & IQ=interquartile range and mean ±SD. (*) *P*<0.05 is considered statistically significant. Group C (standard enteral nutrition) and Group L (fish-oil-based intravenous lipid emulsion). Na (serum sodium), K (serum potassium). AST (Aspartate transaminase), ALT (Alanine transaminase)
